# Cross-Linguistic Data Formats, advancing data sharing and re-use in comparative linguistics

**DOI:** 10.1038/sdata.2018.205

**Published:** 2018-10-16

**Authors:** Robert Forkel, Johann-Mattis List, Simon J. Greenhill, Christoph Rzymski, Sebastian Bank, Michael Cysouw, Harald Hammarström, Martin Haspelmath, Gereon A. Kaiping, Russell D. Gray

**Affiliations:** 1 Department of Linguistic and Cultural Evolution, Max Planck Institute for the Science of Human History, Jena, Germany; 2 ARC Centre of Excellence for the Dynamics of Language, Australian National University, Canberra, Australia; 3 Research Center Deutscher Sprachatlas, Philipps University Marburg, Marburg, Germany; 4 Department of Linguistics and Philology, Uppsala University, Uppsala, Sweden; 5 Department of English Studies, Leipzig University, Leipzig, Germany; 6 Centre for Linguistics, Leiden University, Leiden, Germany; 7 School of Psychology, University of Auckland, Auckland, New Zealand

**Keywords:** Databases, Social anthropology, Interdisciplinary studies

## Abstract

The amount of available digital data for the languages of the world is constantly increasing. Unfortunately, most of the digital data are provided in a large variety of formats and therefore not amenable for comparison and re-use. The Cross-Linguistic Data Formats initiative proposes new standards for two basic types of data in historical and typological language comparison (word lists, structural datasets) and a framework to incorporate more data types (e.g. parallel texts, and dictionaries). The new specification for cross-linguistic data formats comes along with a software package for validation and manipulation, a basic ontology which links to more general frameworks, and usage examples of best practices.

## Introduction

The last two decades have witnessed a dramatic increase in language data, not only in form of monolingual resources^1^ for the world’s biggest languages, but also in form of *cross-linguistic datasets* which try to cover as many of the world’s languages as possible. Creating datasets in linguistics is currently *en vogue*, and apart from traditional ways of linguistic data collection in form of etymological dictionaries, user dictionaries, and grammatical surveys, data are now being published in form of *online databases* (the most complete list of such databases is curated at http://languagegoldmine.com/) and *online appendices or supplements to published papers*, addressing topics as diverse as cross-linguistic lexical associations (cf. http://clics.lingpy.org and http://clics.clld.org), etymologically annotated word lists for large language families like Austronesian (cf. https://abvd.shh.mpg.de^2^ and http://www.trussel2.com/acd/) and Indo-European (cf. http://ielex.mpi.nl), inventories of speech sounds (cf. http://phoible.org), or grammatical features compared across a large sample of the world’s languages (cf. http://wals.info). Along with the increase in the amount of data, there is also an increased interest in linguistic questions, with scholars from both linguistic and non-linguistic disciplines (e.g. archaeology, anthropology, biology, economics, and psychology) now trying to use linguistic data to answer a wide variety of questions of interest to their disciplines. For example, large-scale cross-linguistic studies have recently been conducted to test how robustly languages are transmitted^3^ and which forces drive change^4,5^. Cross-linguistic data have proven useful to detect semantic structures which are universal across human populations^6^, and how semantic systems like color terminology have evolved^7,8^. Another group of studies have analysed cross-linguistic data using quantitative phylogenetic methods to investigate when particular language families started to diverge^9–12^. Cross-linguistic studies have even explored proposed non-linguistic factors shaping languages from climate^13,14^, to population size^15–17^, to genes^18,19^, and how these factors may or may not shape human social behavior at a society level^20^, (All URLS mentioned in this paragraph were accessed July 26, 2018).

Despite this gold rush in the creation of linguistic databases and their application reflected in a large number of scholarly publications and an increased interest in the media, linguistic data are still far away from being “FAIR” in the sense of Wilkinson *et al.*^21^: Findable, Accessible, Interoperable, and Reusable. It is still very difficult to *find* particular datasets, since linguistic journals often do not have a policy on supplementary data and may lack resources for hosting data on their servers. It is also often difficult to *access* data, and many papers which are based on original data are still being published without the data^1^ and having to request the data from the authors is sometimes a more serious obstacle than it should be^22,23^. Due to idiosyncratic formats, linguistic datasets also often lack *interoperability* and are therefore not *reusable*.

Despite the large diversity of human languages, often linguistic data can be represented by very simple data types which are easy to store and manipulate. Word lists and grammatical surveys, for example, can usually be represented by triples of *language*, *feature*, and *value*. The simplicity, however, is deceptive, as there are too many degrees of freedom which render most of the data that have been produced hard to compare. Due to the apparently simple structure, scholars rarely bother with proper serialization, assuming that their data will be easy to re-use. Although there are recent and long-standing standardization efforts, like the establishment of the *International Phonetic Alphabet* (IPA) as a unified alphabet for phonetic transcription^24^, which goes back to the end of the 19th century^25^, or the more recent publication of reference catalogues for languages^26^ and word meanings^27^, linguists often forgo these standards when compiling their datasets and use less strictly specified documentation traditions.

**Figure 1:**
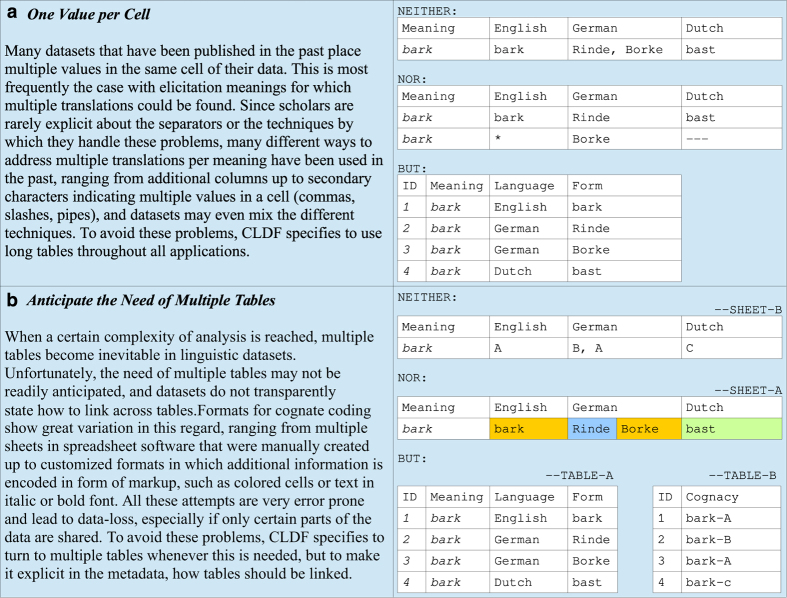
Basic rules of data coding, taking cognate coding in wordlists as an example. (**a**) Illustrates why long tables^53^ should be favored throughout all applications. (**b**) Underlines the importance of anticipating multiple tables along with metadata indicating how they should be linked^44^.

While certain standards, such as the usage of unified transcription systems, are generally agreed upon but often not applied (or mis-applied) in practice, other types of linguistic data come along with a multitude of different standards which make data interoperability extremely difficult (see Fig. 1 for examples on different practices of *cognate coding in wordlists* in historical linguistics).

At the same time, funding agencies such as the *German Academic Research Council* emphasize that ‘the use of open or openly documented formats [to enable] free public access to data deriving from research should be the norm’^28^, mirroring the European Research Council’s guidelines for *Open Access to Research Data* in the *Horizon 2020* programme^29^. Since the importance of cross-linguistic data is constantly increasing, it is time to re-evaluate and improve the state of standardization of linguistic data^30^.

While we have to ask ourselves whether adding another standard might worsen the situation^31^, it is also clear that the current problems of “data-FAIR-ness” in comparative and typological linguistics persist and that standardization is the only way to tackle them. What may set our attempt apart from previous trials is a focus on data re-use scenarios as motivating use cases.

Previously, the focus of standardization attempts was often on comprehensiveness (cf. the GOLD ontology http://linguistics-ontology.org/, accessed July 27, 2018) which led to problems with adoption. Our proposal is more modest, targeting mainly the specific case of tool-based re-use (i.e. analysis, visualization, publication, etc.) of linguistic data. While this may seem overly specific, it is central to the scientific method and reproducible research^32^. This approach may also be particularly successful, because it puts the burden of early adoption on a sample of the linguistics community which may be best equipped to deal with it: the computationalists. The line between computational and non-computational linguists is diffuse enough for the former to act as catalysts for adoption, in particular because tools which can be built on standardized cross-linguistic data include web applications to make data publicly accessible to speaker communities and the general public (cf. http://clld.org, accessed July 27, 2018).

## Results

To address the above-mentioned obstacles of sharing and re-use of cross-linguistic datasets, the *Cross-Linguistic Data Formats* initiative (CLDF) offers modular specifications for common data types in language typology and historical linguistics, which are based on a shared data model and a formal ontology.

### Data Model

The data model underlying the CLDF specification is simple, yet expressive enough to cover a range of data types commonly collected in language typology and historical linguistics. The core concepts of this model have been derived from the data model which was originally developed for the *Cross-Linguistic Linked Data project* (cf. http://clld.org, accessed July 27, 2018), which aimed at developing and curating interoperable data publication structures using linked data principles as the integration mechanism for distributed resources. The CLLD project resulted in a large number of online datasets which provide linguists with a uniform “look-and-feel” despite their diverse content (see Table 1).

The main entities in this model are: (a) *Languages* - or more generally *languoids* (cf. http://glottolog.org, accessed July 27, 2018), which represent the objects under investigation; (b) *Parameters*, the comparative concepts^33^, which can be measured and compared across languages; and (c) *Values*, the “measurements” for each pairing of a language with a parameter. In addition, each triple should have at least one (d) *Source*, as cross-linguistic data are typically aggregated from primary sources which themselves are the result of language documentation based on linguistic fieldwork. This reflects the observation of Good and Cysouw^34^ that cross-linguistic data deal with *doculects*, i.e. languages as they are documented in a specific primary source - rather than languages as they are spoken directly by the speakers.

In this model, each *Value* is related to one *Parameter* and one *Language* and can be based on multiple *Sources*. The many-to-many relation between *Value* and *Source* is realized via *References* which can carry an additional *Context* attribute, which is typically represented by page numbers when dealing with printed sources.

### The CLDF Specification

CLDF is a package format, describing various types of cross-linguistic data; in other words, a CLDF dataset is made up by a set of data files (i.e. files holding tabular data, or tables) and a descriptive file, wrapping this set and defining relations between tables. Each linguistic data type is modeled via a CLDF *module*, with additional, orthogonal aspects of the data modeled as CLDF *components*. “Orthogonal” here refers to aspects of the data which recur across different data types, e.g. references to sources, or glossed examples. This approach mirrors the way Dublin Core metadata terms (a common way of describing metadata, cf. http://dublincore.org, accessed July 27, 2018) are packaged into meaningful sets using *Application Profiles* (cf. http://dublincore.org/documents/2009/05/18/profile-guidelines/, accessed July 27, 2018): a well known technique to support custom, modular - yet interoperable - metadata specifications devised by the Dublin Core Metadata Initiative. CLDF modules are profiles of cross-linguistic data types, consisting of CLDF components and terms from the CLDF ontology.

### CLDF Ontology

The CLDF specification recognizes certain objects and properties with well-known semantics in comparative linguistics. These are listed in a “vocabulary” or “ontology” (cf. https://www.w3.org/standards/semanticweb/ontology for a description of vocabularies in the context of the Semantic Web) - the CLDF Ontology - thereby making them available for reference by URI - the key mechanism of the Semantic Web (that is, the “Web of Data”, cf. https://www.w3.org/standards/semanticweb/data). Wherever possible, this ontology builds on existing ontologies like the *General Ontology for Linguistic Description* (cf. http://linguistics-ontology.org/, accessed July 27, 2018). In particular, the CLDF Ontology makes it easy to link entities in a CLDF dataset to a reference catalogue by providing corresponding reference properties.

### Basic Modules in CLDF

Currently, CLDF defines two modules which handle the most basic types of data which are frequently being used, collected, and shared in historical linguistics and typology (cf. http://clld.org/datasets.html). The *Wordlist* module handles lexical data which are usually based on a *concept list* that has been translated into a certain number of different languages, wich are often further analysed by adding information on cognate judgments or by further aligning the cognate words^35^. The *StructureDataset* module handles grammatical features in a very broad sense, which are usually collected to compare languages typologically.

Two more modules are in an early stage of standardisation: The *ParallelText* module can be used to encode texts which were translated into different languages and are split into functional units (like similar sentences or paragraphs) to render them comparable. The *Dictionary* module makes it possible to encode the lexicon of individual languages.

While these modules are usable in this stage as well, they also serve as examples of the extensibility of the standard: CLDF is intended as iterative, evolving standard, providing a short feedback loop between standardization, implementation and non-standard extensions - thus allowing new data types to be integrated easily.

Each of the modules defines additional components which define relations among the values across languages, inside a language, or value-internally.

### Components

CLDF modules can include *components*. *Components* are pre-defined tables or custom, that is non-standardized, tables. While *components* can have different interpretations, depending on the *module* they are combined with, in the *Wordlist* module they are typically interpreted as concepts and in the *StructureDataset* module they most often interpreted as categorical variables.

### Package Format of CLDF

**Figure 2:**
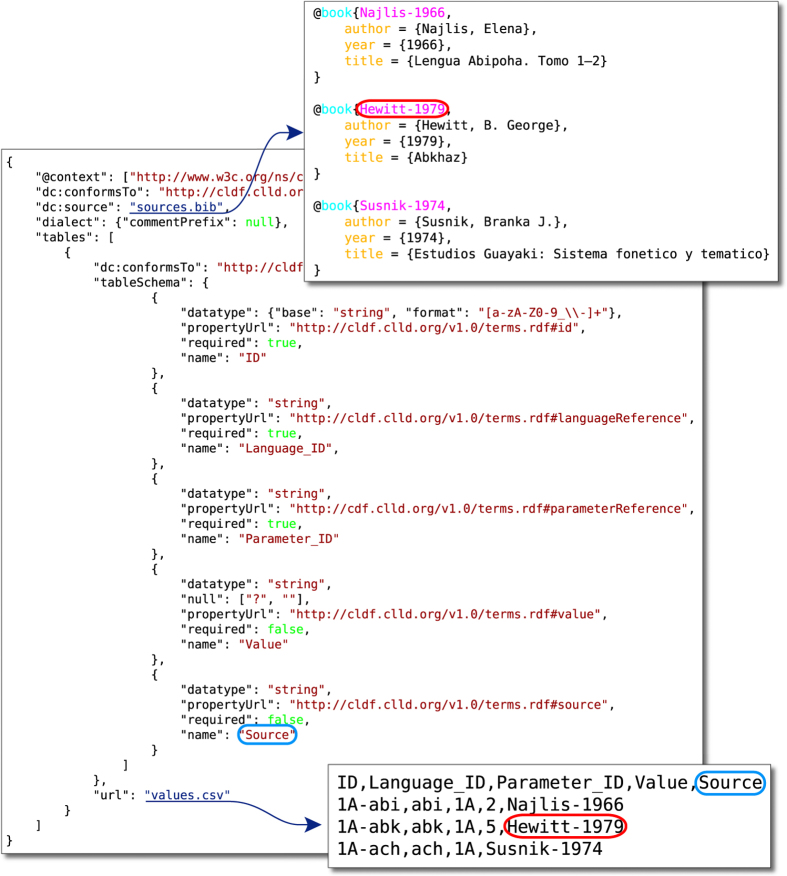
Using CSVW metadata to describe the files making up a CLDF dataset.

CLDF is built on the World Wide Web Consortium (W3C) recommendations *Model for Tabular Data and Metadata on the Web* (cf. https://www.w3.org/TR/tabular-data-model/, accessed July 27, 2018) and *Metadata Vocabulary for Tabular Data* (cf. https://www.w3.org/TR/tabular-metadata/, accessed July 27, 2018, henceforth referred to as CSVW for “CSV on the Web”), which provide a package format allowing us to tie together multiple files containing tabular data (see Fig. 2). Thus, each CLDF dataset is described by a JSON (Javascript Object Notation, see http://json.org/) metadata file according to CSVW tabular metadata specification.

This means that there are standard ways of including metadata: *Common properties* on *table* or *table group* descriptions can be used to add (a) bibliographic metadata using terms from the Dublin Core namespace (cf. http://purl.org/dc/terms/), (b) provenance information using terms from the PROV namespace (cf. https://www.w3.org/ns/prov), (c) catalogue information using terms from Data Catalog Vocabulary (cf. http://www.w3.org/ns/dcat#). Thus, by providing a way to specify such metadata in a machine-readable way, CLDF complements the efforts of the RDA Linguistics Interest Group (cf. http://site.uit.no/linguisticsdatacitation/austinprinciples, accessed July 27, 2018).

### Extensibility of CLDF

The CLDF specification is designed for extensibility. A CLDF dataset can comprise any number of additional tables (by simply adding corresponding table definitions in the metadata file), or by adding additional columns to specified tables. Thus, we expect to see further standardization by converging usage, much like Flickr machine tags evolved (cf. https://www.flickr.com/groups/api/discuss/72157594497877875, accessed July 27, 2018). A dataset may, for example, specify scales for its parameters to guide appropriate visualization. If more and more users employ this new specification, it will become a candidate for standardization within the CLDF specification.

As an example for future enhancement, CLDF could build on extensive metadata schemes like the COREQ standards for qualitative social science research^36^ to allow for an explicit annotation of basic attributes related to language informants when handling original fieldwork data (such as age, gender, multilingualism, etc.). In a similar way, existing semantic web ontologies could be further integrated into the CLDF specification, provided adapters of CLDF find them useful and important.

This extension mechanism (and backwards compatible, frequent releases) allows us to start out small and focused on a handful of use cases and data types for which there is already tool support.

### Reference Catalogues

Creating a lean format like CLDF has been made easier by using reference catalogues to specify entities like languages or concepts. This, in turn, is made possible by employing the linking mechanism built into the W3C model and by leveraging JSON-LD, a JSON serialization of the RDF model underlying the Linked Data principles (cf. https://www.w3.org/TR/json-ld/, accessed July 26, 2018).

Linking to the corresponding properties in the CLDF Ontology allow for unambiguous references to standard catalogues like Glottolog and ISO 639-3^26^ for languoids and Concepticon for lexical concepts. While Glottolog is now well-established among linguists concentrating on cross-linguistic language comparison, Concepticon is a rather young attempt to standardize the reference to lexical concepts as they can be encountered in numerous questionnaires that scholars use in fieldwork and comparative studies. Similar to Glottolog, Concepticon offers unique identifiers for currently 3144 lexical concepts, along with definitions and additional metadata. The lexical concepts defined by Concepticon, however, are not meant to reflect concepts that are expressed by the words in any specific language, but instead link to various resources (so-called *concept-lists*) in which these concepts were elicited. Similar to language names, which show many different variants in the linguistic literature, the glosses which scholars use to elicit a certain concept in cross-linguistic studies may also drastically vary. Linking these elicitation glosses to the Concepticon thus allows for a rapid aggregation of highly diverse datasets. As an example, consider the recently published new version of the CLICS database (cf. http://clics.clld.org), providing information on recurring polysemies for more than 1500 concepts, in which currently 15 different datasets have been aggregated with help of Glottolog and Concepticon. We are currently working on additional reference catalogues for phonetic transcriptions (*Cross-Linguistic Transcription Systems*, cf. https://github.com/cldf/clts, accessed July 27, 2018) and grammatical features (working title *Grammaticon*,^37^) and hope to make them available to CLDF data descriptions by providing corresponding reference properties in future versions of the CLDF Ontology.

However, while including reference properties for certain catalogues facilitates data aggregation and re-use, the CLDF specification does not require the use of any or all reference catalogues. Instead, users should decide what is most applicable to the dataset itself.

### Interacting with CLDF Datasets

The main goal of CLDF is connecting cross-linguistic data and tools. The constituent file formats of CLDF - CSV, JSON and BibTeX -- enjoy ample support for reading and writing on many platforms and in many computing environments. Thus, reading and writing CLDF dataset should be easily achieved in any environment. A sufficiently standardized data format like CLDF means that general data editing tools (e.g. https://visidata.org/) can be used for working with CLDF data (see https://csvconf.com for more information about CSV in science, accessed July 26, 2018). A standardized format allows the community to move from ad-hoc tools programmed by a proficient minority for their particular use case, towards more and better applications, making their functionality available also to researchers without programming skills.

A few such tools already exist. LingPy (cf. http://lingpy.org, accessed July 27, 2018), a suite of open source Python modules, provides state-of-the-art algorithms and visualizations for quantitative historical linguistics; BEASTLing^38^, a Python package, translates human-readable descriptions of phylogenetic inference into the complex driver files for the popular BEAST software; EDICTOR^39^, a graphical JavaScript application, allows scholars to edit etymological dictionary data in a machine- and human-readable way. While the development on these examples began before the CLDF standard, all three of them were originally using CSV dialects for easy data exchange and are now in the process of adding support for CLDF data, thus showing the value of interoperability.

Further, CLDF is standardised such that scripts can easily become shareable and reusable tools for other researchers, rather than one-use scripts. To collect and publish such tools, we initiated a GitHub repository called the CLDF Cookbook (cf. https://github.com/cldf/cookbook). Currently, the cookbook contains recipes for visualization of CLDF datasets, for reading and writing data in CLDF-format from within the LingPy library, and for accessing CLDF data from R.

### A Python API: pycldf

In many research disciplines the Python programming language has become the de-facto standard for data manipulation (often including analyses^40^,). Thus, providing tools for programmatic access to CLDF data from Python programs increases the usefulness of a format specification like CLDF. We implemented a Python package pycldf (cf. https://github.com/cldf/pycldf, accessed July 27, 2018), serving as reference implementation of the CLDF standard, and in particular supporting reading, writing and validating CLDF datasets (cf. https://github.com/cldf/pycldf/tree/master/examples, accessed July 26, 2018).

By making use of the table descriptions in a CLDF metadata file, pycldf can do a lot more. For example, based on the datatype descriptors and foreign key relations specified in table schemas, pycldf can provide a generic conversion of a CLDF dataset into an SQLite database; thereby allowing analysis of CLDF datasets using SQL - one of the work horses of data science. Another example for the usefulness of programmatic access to CLDF data is validation. Having a Python library available for CLDF means validation can be built into LibreOffice’s spreadsheet application or easily run via continuous integration services like Travis on datasets hosted in public repositories (see, for example, https://github.com/lexibank/birchallchapacuran, accessed July 26, 2018).

## Discussion

At the beginning of the CLDF initiative we developed a list of practitioner requirements for cross-linguistic data, based on the experiences of linguists who have worked and are regularly working with cross-linguistic datasets. These practical principles are summarized in Table 2^41^, and when comparing them with our first version of CLDF, it can be seen that CLDF still conforms to all of them. Furthermore, when comparing our initial requirements with the criteria for file formats and standards put forward in guidelines for research data management such as the ones proposed by the WissGrid project^42^, one can also see that the perspectives are largely compatible, thus corroborating our hope that while being sufficiently specific to be of use for linguists, CLDF will also be generic enough to blend in with current best practices for research data management across disciplines.

Following a similar line of reasoning as Gorgolewski *et al.*^43^ lay out in their proposal of a unified data structure for brain imaging data, and building on recommendations from the “Good Practices of Scientific Computing” by Wilson *et al.*,^44^ we decided to base CLDF on well-known and well-supported serialization formats, namely CSV and JSON, with their specific shortcomings being outbalanced by building on CSVW, including its concept of CSV dialects, which allows us to support more variation in tabular data files and help with adaptation of the format. CSVW and its support for foreign keys between tables also allows us to seamlessly implement the recommendation to “anticipate the need to use multiple tables, and use a unique identifier for every record”^43^.

Since CSVW is specified as a JSON-LD dialect (i.e. grounded in the Resource Description Framework RDF, cf. https://www.w3.org/TR/rdf11-primer/, accessed July 27, 2018), it can be combined with an RDF *Vocabulary* or *Ontology* to provide (a) the syntax of a relational serialization format via CSVW, as well as (b) the semantics of the entities in the data model via the ontology. Thus, the CLDF Ontology provides answers to the two questions of “Which things do exist?” and “Which things are based on others?”, which are considered crucial to assess the identification needs for data collections^42^.

Being able to build on Linked Data technologies to attach custom semantics to CSV data is the main advantage for us of CSVW over the similar *Data Package* Standard (cf. https://frictionlessdata.io/specs/data-package/), with its pure JSON package descriptions. It should also be noted that the overlap between these two data packaging specifications is so big and the specifications so similar, that the authors of the *Data Package* standard “imagine increasing crossover in tool and specification support”^45^.

When adopting CSVW as the basis of the specification, it may seem counter-intuitive to model source information via BibTeX - rather than as just another CSV table, linked to with foreign keys. But given that (a) Glottolog - the most extensive bibliography of language descriptions - disseminates BibTeX and (b) the many-to-many relation between values and sources would have required an additional association table, (c) BibTeX is a standard format readable and usable by most citation software programs, BibTeX seemed to be the right choice when maximizing maintainability of datasets.

Another design decision taken with CLDF was to not specify a single-file format. Instead of forcing users to provide their data in database formats, like SQLite (cf. https://sqlite.org/appfileformat.html, accessed July 27, 2018), or in pure text formats with extensible markup, like the NEXUS format in biology^46^, we opted for specifying a multi-file format - and deliberately chose to not define any packaging. Instead, we regard packaging of usually rather small sets of small text files as a problem for which multiple solutions with particular use cases have already been proposed (e.g. *zip* for compression, *bagit* for archiving, etc., cf. https://tools.ietf.org/html/draft-kunze-bagit-14, accessed July 27, 2018). We do not even have to specify a particular directory layout for the multiple files forming a CLDF dataset, because the description file references data files using URIs, thereby turning CLDF into a multi-file format almost as flexible as HTML. While this decision goes against the idea of “self-describing data” - underlying formats like XML - it works well with databases with established curation workflows, because it provides an inobtrusive way to enhance the existing dataset: For example the “traditional” WALS Online tab-separated format (e.g. http://wals.info/feature/1A.tab) can be turned into a CLDF dataset (by anyone) by providing a separate description file, just referencing the tab-separated file as data file.

Since CLDF has been developed in close collaboration with researchers working on different ends of data-driven research in historical linguistics and language typology, CLDF is already being used by large linguistic projects (cf. http://clics.clld.org/ and http://www.model-ling.eu/lexirumah/, both accessed July 27, 2018) and as the data format for publishing supporting information^11,47^. CLDF is the native format for the forthcoming global language databases *Grambank*, *Lexibank* and *Parabank* (cf. http://glottobank.org/) being developed by a consortium of research centers and universities. Further, CLDF is by now already supported by a larger number of software packages and applications, ranging from libraries for automatic sequence comparison in historical linguistics (LingPy), via packages for phylogenetic analyses (BEASTLing^38^), up to interfaces for data inspection and curation (EDICTOR^39^).

Since the CLDF initiative was born out of the Cross-Linguistic Linked Data (CLLD) project, it is readily integrated into the CLLD framework and will allow users to publish their data without efforts on the web, making their data *findable* by exposing data and metadata to the major search engines, and increasing thus their interoperability. An important part of enabling data re-use is making data discoverable. In today’s digital environment this means largely being “present” on the web. Basing CLDF on the recommendations of W3C’s *Tabular Data on the Web* working group is a partial answer to this requirement.

Making it simple to publish CLDF datasets as CLLD applications goes a step further, because CLLD applications improve the visibility of datasets by exposing data and metadata to the major search engines, but also to field-specific aggregators such as OLAC, the *Open Language Archives Community*. More specifically, since CLLD applications implement the data provider part of the OAI-PMH protocol (cf. http://www.openarchives.org/OAI/openarchivesprotocol.html, accessed July 27, 2018) a CLDF dataset served by a CLLD application will be discoverable from OLAC and other portals.

It is important to note that CLDF is not limited to linguistic data alone. By embracing reference catalogues like Glottolog which provide geographical coordinates and are themselves referenced in large-scale surveys of cultural data, such as D-PLACE^48^, CLDF may drastically facilitate the testing of questions regarding the interaction between linguistic, cultural, and environmental factors in linguistic and cultural evolution.

## Methods

Efforts to standardize cross-linguistic data, in particular typological datasets and with the aim of comparability across datasets, have been undertaken since at least 2001, when Dimitriadis presented his *Typological Database System*^49^ (cf. http://languagelink.let.uu.nl/tds/index.html, accessed July 27, 2018). One initial step was to introduce general database principles to database design in linguistic typology^50^.

Rather than standardizing data formats, the CLLD project largely tried to standardize the software stack for cross-linguistic databases. Still, the core data model which could be extracted from these database software implementations served as one of the inspirations when standard data formats were discussed at the workshop *Language Comparison with Linguistic Databases*, held 2014 at the Max Planck Institute for Psycholinguistics in Nijmegen.

The followup workshop *Language Comparison with Linguistic Databases 2* - held in 2015 at the Max Planck Institute for Evolutionary Anthropology in Leipzig - saw concrete proposals towards what now is CLDF^41^; and later this year, the workshop *Capturing Phylogenetic Algorithms for Linguistics* - held at the Lorentz Center in Leiden - brought together people interested in analysis of cross-linguistic data, thus providing a test bed for the proposals.

Apart from these larger meetings where scholars discussed ideas of standardization, the CLDF-initiative profited from the numerous Glottobank meetings organized by the Department of Linguistic and Cultural Evolution at the Max Planck Institute for the Science of Human History (Jena), in which big-picture ideas of standards for linguistic data were discussed in more concrete terms by smaller teams which came forward to work on specific aspects of the specification, including reference catalogues like Concepticon, the handling of etymological data, and linking to external projects like D-PLACE.

These events formed a group representing the main institutions in the small field of large-scale comparison of cross-linguistic data, which contributed to the CLDF specification.

When a Linguistics Data Interest Group was endorsed by Research Data Alliance (RDA) in 2017, echoing RDA’s call to ‘develop and apply common standards across institutions and domains to ensure greater interoperability across datasets’ in Linguistics, this coincided nicely with the progress of CLDF 1.0.

### Code Availability

The CLDF specification is curated using a GitHub repository (cf. https://github.com/cldf/cldf). Released versions are published and archived via Zenodo under the Apache 2.0 license. The current version of the specification is CLDF 1.0.1^51^.

The pycldf package is maintained in a GitHub repository (cf. https://github.com/cldf/cldf). Released versions are available from the Python Package Index (cf. https://pypi.python.org/pypi/pycldf) and archived with Zenodo^52^ under the Apache 2.0 license.

## Additional information

**How to cite this article**: Forkel, R. *et al.* Cross-Linguistic Data Formats, advancing data sharing and reuse in comparative linguistics. *Sci. Data*. 5:180205 doi: 10.1038/sdata.2018.205 (2018).

**Publisher’s note**: Springer Nature remains neutral with regard to jurisdictional claims in published maps and institutional affiliations.

## Figures and Tables

**Table 1:** Examples of popular databases produced within the CLLD framework.

**Name**	**URL**	**Description**
World Atlas of Language Structures	wals.info	Grammatical survey of more than 2000 languages world-wide.
Comparative Siouan Dictionary	csd.clld.org	Etymological dictionary of Siouan languages.
Phoible	phoible.org	Cross-linguistic survey of sound inventories for more than 2000 languages world-wide.
Glottolog	glottolog.org	Reference catalogue of language names, geographic locations, and affiliations.
Concepticon	concepticon.clld.org	Reference catalogue of word meanings and concepts used in cross-linguistic surveys and psycholinguistic studies.

**Table 2:** Practical demands regarding cross-linguistic data formats.

**Abbr.**	**Requirement**	**Note**
P	PEP 20	“Simple things should be simple, complex things should be possible” (cf. https://www.python.org/dev/peps/pep-0020/, accessed July 27, 2018): Datasets can be one simple CSV file, encoding language-parameter-value-triples.
R	Referencing	If entities and parameters can be linked to reference catalogues such as Glottolog or Concepticon, this should be preferred to duplicating information.
A	Aggregability	Data should be simple to concatenate, merge, and aggregate in order to guarantee their reusability.
H	Human- and machine-readability	Data should be both editable *by hand* and amenable to reading and writing by software (preferable software which typical linguists can be expected to use).
T	Text	Data should be encoded as UTF-8 text files or in formats that provide full support for UTF-8.
I	Identifiers	Identifiers should be resolvable HTTP-URLs, where possible, if not, this should be documented in the metadata.
C	Compatibility	Compatibility with existing tools, standards, and practices should always be kept in mind and never easily given up.
E	Explicitness	One row should only store one data point, and each cell should only have one type of data, unless specified in the metadata.
